# The role of environmental attitudes and consumption patterns in consumers’ preferences for sustainable food from circular farming system: a six EU case studies

**DOI:** 10.1186/s40100-025-00350-0

**Published:** 2025-02-19

**Authors:** Selene Ivette Ornelas Herrera, Yasmina Baba, Zein Kallas, Erik Meers, Evi Michels, Zoltán Hajdu, Ana Marija Spicnagel

**Affiliations:** 1 Centre for Research in Economy and Agrofood Development - Polytechnic University of Catalonia (CREDA- UPC), Parc Mediterrani de La Tecnologia. Edifici EEABB, Esteve Terrades 8, 08860 Castelldefels, Barcelona, Spain; 2 Barcelona University (UB), Barcelona, Spain; 3 Ghent University (UGENT), Ghent, Belgium; 4 Soltub Trade and Service Providing Limited Liability (SOLTUB), Budapest, Hungary; 5 Konzalting Doo Za Poslovne Usluge (IPS), Zagreb, Croatia

**Keywords:** Consumer preference and willingness to pay, Consumption behaviour, Environmental attitudes, Sustainable production, Circular food products, Labelled discrete choice experiment

## Abstract

Decisions about what we consume have environmental repercussions and, therefore, implications for future generations. Consumers’ ability to pay more for sustainable food stimulates production strategies, like circular agriculture, aiding the European Green Deal’s sustainable food system. With the aim of analysing the preferences and willingness to pay of European consumers for food labelled as obtained by more sustainable systems in terms of reduced greenhouse gas emissions, and the optimisation of soil nutrients, a survey with 5591 participants from Spain, Poland, Italy, Hungary, Croatia, and Belgium was conducted. The survey was designed to analyse three food products (pork meat, milk, and bread) obtained through different production systems (circular, conventional, and organic) using the discrete choice experiment methodology. The survey included questions about consumers’ environmental attitudes and consumption behaviour, to identify their influence over preferences regarding sustainable food products. Results revealed that over 27% of consumers preferred circular food, unveiling a potential market. This preference highlighted the effect of consumers’ environmental attitudes. Those who actively engaged in recycling were more prone to choose circular food and shown a tendency to go for less conventional options. Consumers’ WTP was consistently higher for circular milk compared to conventional across all the studied countries. However, for circular pork and bread, this greater WTP was reported exclusively in Spain and Croatia. It was suggested that sectors involved in a sustainable food production should standardise labels for circular food, create educational programmes about problems generated by unsustainable consumption, and promote consumption of circular products.

**Supplementary Information:**

The online version contains supplementary material available at 10.1186/s40100-025-00350-0.

## Introduction

The sustainability of the food system is a complex and multidimensional concept that involves not only producers, policymakers, and researchers, but also consumers (Nguyen 2018). Current production and consumption decisions have clear societal and environmental impacts, and compromise the welfare of future generations (Baranowski 2023; FAO 2017). Through the European Green Deal, the European Commission aims to boost a more sustainable food system, targeting both production and consumption by ensuring a fair price to farmers and consumers, promoting healthy and environmentally friendly food products that address climate change and environmental challenges (Bock et al. 2022). Within the Green Deal strategy, the farm-to-fork strategy was prioritised. In it, several action plans were designed to improve food system sustainability through a legislative framework targeting food production, processing, retail, consumption, and food loss and waste (Fetting 2020). In this context, the circular economy model was proposed as a main action plan, whose primary objectives are ensuring less waste, empowering consumers and stakeholders, introducing a labelling framework for a sustainable production system in the EU, and including the circularity concept in society activities (Georgios and Nicoleta 2023).

The EU’s voluntary “sustainable consumption pledge” initiative (EU 2020) also represents one of the main actions for empowering consumers. It encourages companies to take a voluntary pledge to support sustainable consumption. The pledge focuses on communicating their environmental footprint to consumers through Ecolabel schemes that highlight circularity in their activities (using more recycled or sustainable sourced materials, reducing waste and energy consumption). The EU agenda reflects the need to consider consumer protection requirements in policy formulation and implementation, and emphasises the need to boost the certification system in the transition towards more sustainable production and consumption models. It is expected to approve a legal sustainable food labelling framework that governs the provision of information to consumers related to the sustainability of food products at the EU level. Regarding the above mentioned, there is a need to understand the current situation of consumer preference for sustainable food systems and circular labelling.

## Literature review

Consumer preferences refer to the choices and willingness to pay (WTP) made by consumers among diverse competing products based on attributes and price. According to Lancaster’s consumer theory, such preferences are influenced by various factors (Raun Mørkbak et al. 2010). The analysis of consumer preferences is based on numerous economic theories that aim to clarify how consumers choose between different goods and services (Hands 2014). The Utility Theory posits that consumers choose alternatives to maximise their utility or satisfaction (Ali-Beshir et al. 2021). The Theory of Planned Behaviour (TPB), proposed by Ajzen (1991), asserts that attitude, subjective norm, and perceived behavioural control affect consumers’ behavioural intentions, with attitude acting as a principal determinant of purchase intentions (Qin and Song 2022). Another important theory is the Values, Beliefs, and Norms (VBN) Theory proposed by Stern et al. (1999). The VBN theory argues that environmental attitudes and perceptions are essential to action strategies. The TPB and VBN theories improve understanding of environmental intents and behaviours, clarifying how positive attitudes towards food products that minimise environmental impacts may lead to a greater likelihood of purchasing sustainable products (López and Sánchez 2012; Ashaduzzaman et al. 2022).

Numerous studies have been undertaken regarding consumer food preferences and their willingness to pay, employing discrete choice experiments to describe the trade-offs consumers consider when selecting food products and the premium they are willing to pay for certain attributes (Lusk and Schroeder 2004; Bliemer and Rose 2011; Dinh et al. 2021; Ellis et al. 2021). These studies analyse various product attributes (including price, origin, production system, and labelling), consumer socio-demographics (such as age, gender, and education), and behaviours and attitudes including environmental attitudes and sustainable consumption patterns (Ali et al. 2021; Li et al. 2021; Muresan et al. 2022; FAO 2022).

The results demonstrated that the production system, that we based on choice study, is the second most significant factor influencing consumers´ preference, following price, as emphasised by Rahmani et al. (2019). In this context, understanding how food is produced is a key factor in understanding consumer preference. Sustainable food production implies a strategy for resource utilisation that considers its ecological and social impacts (Lichtfouse et al. 2009). Circular farming is a sustainable agricultural method that reduces external inputs and incorporates the recycling and repurposing of agricultural by-products and waste as inputs in many different procedures. It is based on a circular economic principle of “grow, produce, use and restore”, rather than following a linear model of “extract, produce, use and dispose” (Helgason et al. 2021; Rauw et al. 2023). It emphasises the closure of nutrient and material cycles, the minimisation of natural resources consumption, the reduction of waste generation, the mitigation of greenhouse gas emissions, and the decrease in water and soil contamination (Tagarakis et al. 2021). Circular farming provides the opportunity to increase production efficiency while maintaining a respectful balance with the environment (Yue et al. 2022) and improving socioeconomic benefits to achieve sustainable agriculture (Li and Kallas 2021). On the other hand, organic farming is an agricultural production system that promotes biodiversity and soil health by eliminating dependence on chemical fertilisers, pesticides, and plastics, as well as antibiotic-free livestock production (Helgason et al. 2021; Rauw et al. 2023; Rao et al. 2023). Conversely, conventional farming is an intensive production system that employs a linear production model. It includes conventional agricultural practices that emphasise productivity and increased yields through the use of new technologies and the widespread application of synthetic fertilisers (Morgan and Murdoch 2000). While organic and circular food production systems are recognised as more sustainable agricultural practices, it is important to understand consumers’ preferences and attitudes towards these sustainable systems in comparison with conventional ones.

In this context, several studies found also that environmental attitudes are related to consumer preferences and WTP a premium for more environmentally friendly food products (Ayub et al. 2020; Kushwah et al. 2019), such those obtained from circular farming. As a result, in many cases, consumption patterns are strongly influenced by consumers’ environmental attitudes, and their preferences for sustainability (Dueñas Ocampo et al. 2014). As consumers become more eco-aware (“ecocentric environmental attitude”) and learn more about how food production affects the environment, they become more interested in purchasing food that comes from more sustainable farming methods, like circular farming proposed in our study (Di Santo et al. 2024; Ornelas et al. 2024).

Recent researches have studied consumer preferences towards food products produced from circular economy concepts, notably those made from by-products or waste. These studies indicate that consumers exhibit a willingness to pay for such products (McCarthy et al. 2019; Coderoni and Perito 2020) and are motivated mainly by environmental concerns (Lorek and Spangenberg 2014). Furthermore, these studies revealed several trends: Younger with high education level consumers are generally inclined towards circular economy principles in the food sector (Fogarassy et al. 2020; Ali et al. 2021; Ornelas et al. 2024; Di Santo et al. 2024). Providing information about environmental benefits and production processes can enhance consumer acceptance (Hellali et al. 2023; Borrello et al. 2017). However, overall awareness and understanding of circular economy concepts in food remain low among consumers (Sousa et al. 2021). Women tend to show more favourable attitudes towards sustainable foods (Ureña et al. 2008; Sivathanu 2015) and are more concerned about the impact of food waste on social equity and family budgets (Cantaragiu 2019). Higher education levels are associated with increased interest in sustainable and healthier food options (Ovsyannikov 2020; Fraser et al. 2000). Consumer education from credible sources is crucial for creating awareness of circular food consumption (Fox et al. 2023). In this context, results showed that circular innovation can effectively target highly educated young individuals with high incomes, Fogarassy et al. (2020). Financial constraints significantly affect consumer preferences and decision-making, with financially restricted individuals prioritising material possessions over experiences, Tully et al. (2015). To advance circular food systems, it is essential to implement targeted marketing strategies and education campaigns that emphasise environmental and human benefits, as well as sustainability (McCarthy et al. 2019; Cattaneo et al. 2019; Fogarassy et al. 2020).

This paper contributes to the literature on consumers´ preferences by providing a deep understanding of consumers’ attitudes and preferences towards circular farming as a sustainable agricultural system. This study is among the few that compare “circular” farming, incorporating the circular economy concept, with current organic and conventional practices by evaluating consumers’ willingness to pay a premium for “circular” food products. It also determines whether there is a market potential for circular food products, which helps businesses understand the demand and price marketing strategies if launched at marketplace. Furthermore, it provides valuable results that policymakers can use to promote the adoption of circular farming as a sustainable agricultural practice, demonstrating farmers that there is a market potential, positive consumer preferences towards the development of circular economies at farm level. This study also contributes to the assessment of circular farming’s economic viability by incorporating the private benefit obtained from evaluating consumer willingness to pay a premium for circular bread, milk, and pork compared to conventional and organic systems, which may assist policymakers in developing more efficient agricultural policies to promote the adoption of such circular innovations at the farm scale. Consumers play a crucial role in driving the development of circular business models and promoting sustainable practices in various industries, including agriculture. Their preferences and choices significantly influence the adoption of circular agronomic practices (Lopes et al. 2023).

## Materials and methods

### Survey structure and data collect

The DCE was used to analyse consumers’ preferences and WTP towards three product categories: pork for pig production, milk for cattle production, and bread for cereal production. The products were selected taking into account that: The EU is the second-biggest producer of pork in the world (European Commission 2018), and pork meat is part of the principal carbon footprint emitters (Poore and Nemecek 2018). The dairy sector is the second-biggest agricultural sector in the EU, representing more than 12% of agricultural output (European Parliament 2018), and milk production contributes 3–4% of global GHG emissions (Laca et al. 2020). The EU harvested production of cereals, including rice, was 297.5 million tonnes in 2021, representing about 11.3% of global production (Eurostat 2021), and represents 31% of total agricultural area utilised in Europe (Ferreira et al. 2021).

Hypothetical markets with several simulated purchasing situations were created following a statistical design for each product category under three farming systems (conventional, organic, and circular). Consumers’ purchasing habits, their sustainable behaviours and actions, such as food waste and recycling habits, were analysed using Likert scales (Molinillo et al. 2020). Consumers’ environmental attitudes were analysed through the New Ecological Paradigm (NEP) scale (Gomera et al. 2013). Additionally, some socio-demographic and economic questions were included (age, gender, employment status, education level, and current financial situation). This methodology was applied to the data of each country and each product separately.

**Fig. 1:**
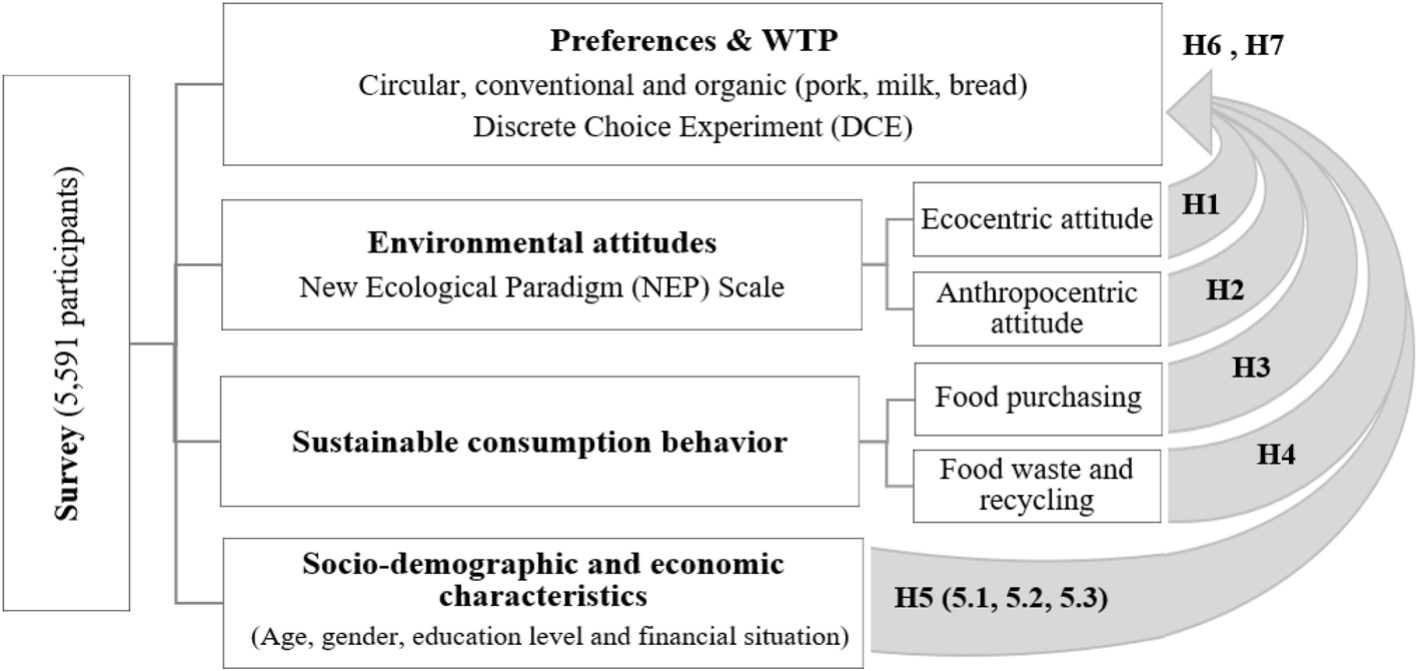
Methodological approach

The questionnaire was approved by the ethical committee in accordance with European regulations (EU regulations 2016/679). Data were collected online in Belgium, Croatia, Hungary, Italy, Poland, and Spain from a total of 5591 participants using the Qualtrics market research company, and Netpanel market company from June 2021 to January 2022, as part of a European project. Each sample was stratified in terms of gender and age to represent the average population in each country. Respondents included in this study were only those that are mainly or in part responsible for household food shopping (Weinrich and Elshiewy 2019). The methodological approach followed is summarised in Fig. 1, in order to gain a holistic view of the research carried out and the different aspects addressed.

As a result of the literature review, the following hypotheses were proposed:

#### H1

Ecocentric environmental attitudes are positively related to a preference for circular food.

#### H2

Anthropocentric environmental attitudes are negatively related to a preference for circular food.

#### H3

Sustainable food purchasing habits are positively related to a preference for food labelled as circular.

#### H4

Sustainable food waste management and recycling habits are positively related to a preference for food labelled as circular.

#### H5.1

The young have a greater WTP for circular food than older consumers.

#### H5.2

Women are likely to pay more for circular food products.

#### H5.3

A good financial situation is positively related to a willingness to pay more for circular food products.

#### H6

The WTP in percentage terms is higher for less expensive products, such as milk or bread, than for meat.

#### H7

Preferences are related to country context.

### The method of measuring consumers’ willingness to pay

The DCE was used to derive consumers’ WTP and purchase intention. The DCE aims to identify the consumers’ trade-offs in their choice decision (Lusk and Schroeder 2004). In the standard application of the DCE, the first step is to identify the main attributes and level that describe the different products. However, a labelled design can be used when the price is the only discriminant attribute across products in choice sets. This kind of designs is helpful when the products are either new or unavailable in the local marketplace and when the decision to purchase is too complex. In this case, consumers are shown different labelled products differentiated only by the price and told to imagine that the products offered in the choice sets are exactly the same as those they usually purchase in terms of other attributes and descriptors (origin, packaging, brand, nutritional values, and certifications).

**Fig. 2:**
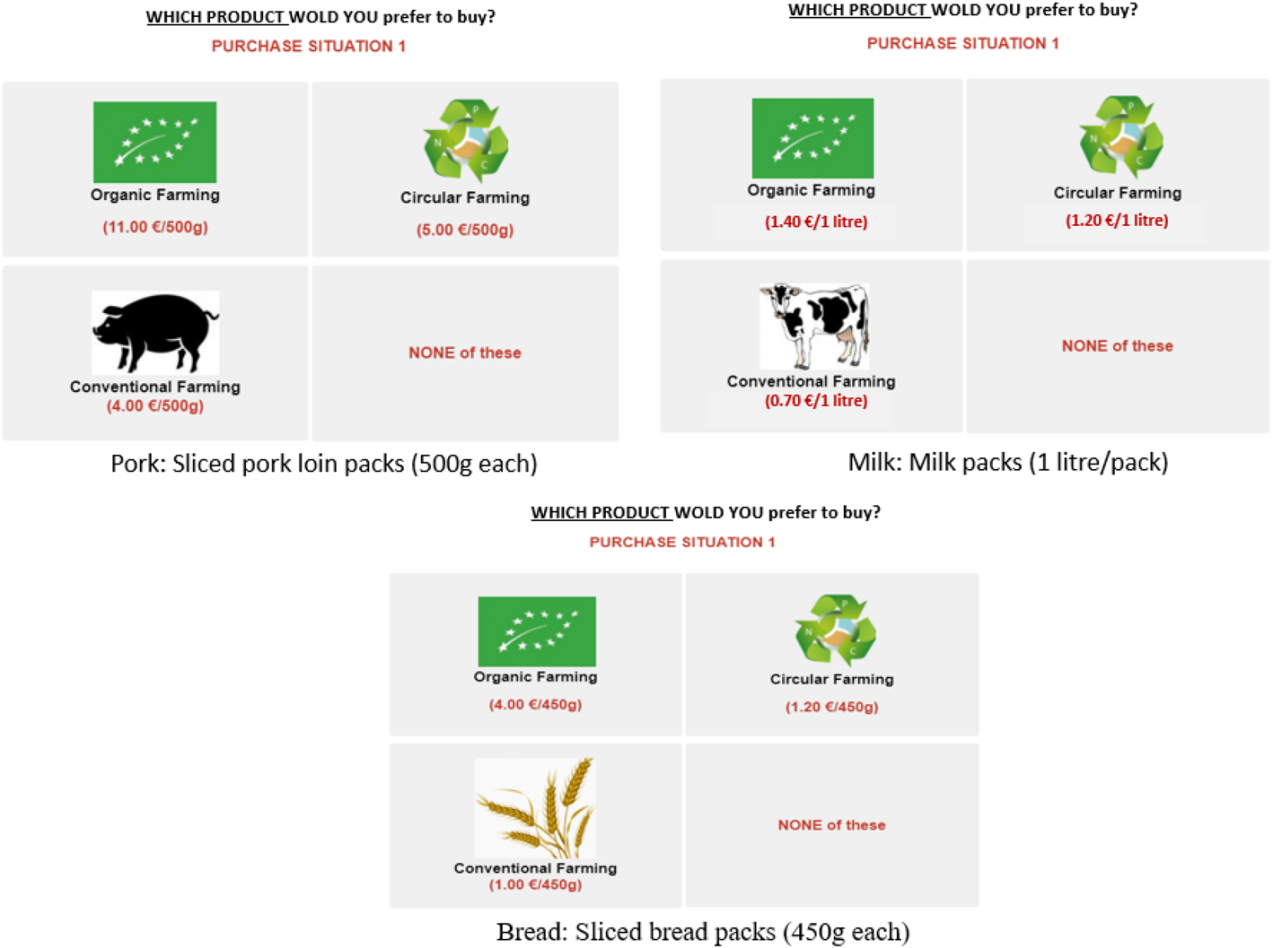
DCE Scenario 1 for each product (pork, milk, and bread), Spain case study

Experiments were designed for each country and selected product. The product category (pork, milk, and bread) was labelled with three different production systems (conventional, organic, and circular) and repeated in all purchasing scenarios at different price combinations using a D-optimal and efficient design defined by the Ngene software 2019. The D-optimal designs increase the statistical performance by minimising the standard errors on parameter estimates (Bliemer and Rose 2011). The choice sets for each product were built by adding to the organic, circular, and conventional alternatives the “NONE” alternative, as an “opt-out” option to be consistent with demand theory and to make the choice task more realistic. Accordingly, four choice sets were obtained for each product by ensuring the orthogonality of prices across the products. The first scenario “choice set” of each product for the Spain case study is shown in Fig. 2, as an example.

The prices vector of each product category and farming system was identified according to the specific products (type and weight): 500 gr sliced loin of pork, 450 gr sliced bread, and 1-L milk. For each product and farming system, four price levels were identified in each case study. Price levels and product size were identified after a deep review and comparison with similar products at market level for each country. In the case of the circular farming systems, the price levels were defined between the interval of the highest prices of conventional and the lowest prices of organic products, because the circular products were not available in the marketplace compared to conventional and organic products. The price vectors for products from circular farming were considered relatively higher than the average price of the conventional alternatives at a real marketplace and lower than that of the organic one. The price vectors for each product category, farming system and country, are shown in Tables 1, 2, and 3.

**Table 1:** Price vectors of pork in each case study

Product	Pork: Sliced pork loin packs (500 g each)					
Price levels (€)	Spain	Poland	Italy	Hungary	Croatia	Belgium
ORG	9.00	5.65	8.00	5.50	6.60	12.00
	10.00	6.30	10.00	6.00	6.90	14.00
	11.00	6.95	12.00	6.50	7.20	16.00
	12.00	7.60	14.00	7.00	7.50	18.00
CONV	3.00	2.20	3.00	3.00	3.30	5.00
	4.00	2.60	3.80	3.20	3.80	6.00
	5.00	3.00	4.60	3.40	4.30	7.00
	6.00	3.40	5.40	3.60	4.80	8.00
CIRC	5.00	3.00	4.60	3.40	4.20	7.00
	6.00	3.50	5.40	3.60	4.70	8.00
	7.00	4.00	6.20	3.80	5.20	9.00
	8.00	4.50	7.00	4.00	5.70	10.00

**Table 2:** Price vectors of milk in each case study

Product	Milk: Milk packs (1 L/pack)					
Price levels (€)	Spain	Poland	Italy	Hungary	Croatia	Belgium
ORG	1.20	0.90	1.50	1.30	1.60	1.40
	1.30	1.70	1.60	1.40	1.75	1.50
	1.40	2.15	1.70	1.50	1.90	1.60
	1.50	2.60	1.80	1.60	2.05	1.70
CONV	0.60	0.45	0.90	0.70	0.50	0.80
	0.70	0.50	1.00	0.80	0.65	0.90
	0.80	0.55	1.10	0.90	0.80	1.00
	0.90	0.60	1.20	1.00	0.95	1.10
CIRC	0.90	0.55	1.10	0.90	0.85	1.00
	1.00	0.60	1.20	1.00	1.00	1.10
	1.10	0.70	1.30	1.10	1.15	1.20
	1.20	0.75	1.40	1.20	1.30	1.30

**Table 3:** Price vectors of bread in each case study

Product	Bread: Sliced bread packs (450 g each)					
Price levels (€)	Spain	Poland	Italy	Hungary	Croatia	Belgium
ORG	2.50	2.80	1.40	1.50	2.10	2.10
	3.00	3.30	1.60	1.60	2.40	2.30
	3.50	3.80	1.80	1.70	2.70	2.50
	4.00	4.30	2.00	1.80	2.90	2.70
CONV	0.80	0.90	0.80	0.90	1.00	1.40
	1.00	1.20	0.90	1.00	1.20	1.50
	1.20	1.50	1.00	1.10	1.40	1.60
	1.40	1.80	1.10	1.20	1.60	1.70
CIRC	1.20	1.50	1.00	1.10	1.40	1.60
	1.40	1.80	1.10	1.20	1.60	1.70
	1.60	2.20	1.20	1.30	1.80	1.80
	1.80	2.50	1.30	1.40	2.00	1.90

Before showing the different scenarios to the participants, they received information about the main characteristics of each production system (livestock and farming)—conventional, organic, and circular—describing how the products were obtained. Appendix 1 of the supplementary material contains descriptions of the production systems.

In order to reduce the impact of hypothetical bias on the results, a cheap talk script was used, and participants were also informed that respondents often overestimate their WTP in hypothetical purchase situations, because they do not consider their food budget constraints (Ellis et al. 2021; Loomis 2014 and Carlsson et al. 2005).

#### Estimating consumers’ WTP: The modelling

The DCE relies on Lancaster’s Theory of Value (Lancaster 1966) and on Thurstone’s Random Utility Theory (Thurstone 1927). According to this theory, individuals choose among the alternatives in a choice situation according to a utility function with two main components: a systematic (observable) component and a random error term (non-observable):

1 $$U_{jn} = V_{jn} + \varepsilon_{jn}$$

where *U*_*jn*_ is the utility of alternative j to subject n, *V*_*jn*_ is the systematic component of the utility, and *ε*_*jn*_ is a stochastic term. Assuming linearity, the utility function for alternative j can be expressed as follows:

2 $$V_{jn} = \beta_{j} \cdot ASC_{j} + \alpha_{j} \cdot P_{jn}$$

where *j* is the ORG, CONV, and CIRC products at the different production systems, *P*_*jn*_ is the price of alternative *j* selected by the consumer *n*, and *β*_*j*_ are the coefficients of the alternative specific constant (ASC) for each alternative *j* relative to the NONE option, which in our case study represents the marginal utility of alternative *j*. *α*_*j*_ are the coefficients representing the effect of the *j*th product price on the utility for the *j*th product.

To predict the subjects’ preferences for an alternative (i.e. a product obtained from a specific production system), we need to define the “probability of choice” that individual n chooses the alternative *i* rather than the alternative *j* (for any *i* and *j* within choice sets, T). McFadden (1974) developed the base model for the DCE often referred to as the multinomial logit (MNL) model. According to this model, the probability that a consumer n chooses production system *j* is

3 $${\text{Prob}}\;\left\{ {j\; {\text{is}} \;{\text{chosen}}} \right\} = \frac{{e^{{\mu \;V_{jn} }} }}{{\mathop \sum \nolimits_{k = 1}^{J} e^{{\mu \;V_{jn} }} }}\;\;\forall k \in T$$

where *μ* is a scale parameter that is inversely related to the variance of the error term.

For the MNL, the scale parameter is fixed to one for estimation reasons. Furthermore, in this model specification, the condition of the Independent and Identically Distributed (IID) error term must be met according to a Gumbel distribution. Such a distribution in the error term allows for the verification of a restrictive property within the MNL, which is the Independence of Irrelevant Alternatives (IIA) property. This restriction implies that the ratio of the probabilities of choosing any pair of alternatives *i* and *j* [(P(i/T))/(P(j/T))] is not dependent on the systematic utility of any other alternative within the set of alternatives, which is seldom ensured. As a consequence, the MNL imposes a very strict structure on cross-price elasticities, avoiding the possibility of analysing substitutability between the products (Hensher et al. 2005). In this context, the universal or the “mother” logit model can be estimated (McFadden et al. 1977) for the labelled type design used in this study. In this model, the utility of each product is specified as a function of the descriptors of the other products. In our specific case studies, the utility of each product is a function of the ASC and the prices of all the other products. For instance, the utility of the ORG is a function of an ASC of the ORG and the prices of the ORG, the CONV, and the CIRC production system of the product.

In this case, the utility function for production system j in the universal logit model is:

4 $$V_{jn} = \beta_{j} \cdot {\text{ASC}}_{j} + \mathop \sum \limits_{k = 1}^{J} \alpha_{jk} \cdot P_{kn}$$

where *j *= the ORG, CONV, and CIRC, k is from 1 to 4 (i.e. the three products presented: the ORG, CONV, and CIRC at 4 price levels, and the NONE option), *P*_kn_ is the *k*th product’s price for consumer n, and *α*_jk_ represents the effect of the *k*th product’s price on the utility of the *j*th product.

To estimate the universal Logit model, Eq. (4) is placed into Eq. (3). However, the estimation of the model following Eq. (3) may clearly incorporate the violation of the independence from the irrelevant alternatives (IIA) assumption commented upon before. Thus, we considered the mixed logit models (MIXL) (also referred to in the literature as the Random Parameter Logit model, RPL), that relaxes the IIA assumption.

The RPL model extends the MNL model by allowing for unobserved heterogeneity through random coefficients on attributes (Ben-Akiva et al. 1997). In our case studies, the random parameters were assigned on the ASC, since this estimate encompasses all descriptors of the product in a holistic way. According to this model, the coefficient vector for person n is $\overline{\beta } + \sigma \lambda_{n}$, where *β ¯* is the estimated mean and *σ* is the standard deviation of the marginal distribution of *β* and *λ*_*n*_ is a random term assumed normally distributed with mean zero and unit standard deviation. Thus, the term *σλ*_*n*_ is the vector of person n specific deviations from the mean value of the *βs*. The *η*_*n*_ is described by an underlying continuous distribution for the attributes (again in our case the ASC). In most applications, the multivariate normal distribution is the most used, MVN (0, Σ). In our case, we considered the ASC independently normally distributed in the population. The price coefficients were considered fixed (i.e. non-random) to ensure that the estimated total WTP distribution was finite. This is because the total WTP of a product j versus the baseline alternative NONE (i.e. none of the presented products) is calculated as the negative ratio of the ASC coefficient to the price coefficient of the same product j (Lusk and Schroeder 2004):

5 $${\text{WTP}}_{{{\text{Product}}\,j \, Vs.{\text{ No - option}}}} = - \left( {\frac{{\frac{d}{{d{\text{ACS}}_{j} }}\beta_{j} \cdot {\text{ASC}}{}_{j}}}{{\frac{d}{{dP_{kn} }}\alpha_{jk} \cdot P_{kn} }}} \right) = - \left( {\frac{{\beta_{j} }}{{\alpha_{jk} }}} \right)\; = \left( {\frac{{\beta_{{{\text{Product }}j}} }}{{\alpha_{{{\text{price }}j}} }}} \right)$$

This calculation relies on the estimation of the Marginal Rate of Substitution (MRS) of any two coefficients. Since one of the coefficients is a monetary one (i.e. the price), it is possible to determine the WTP. The marginal WTP for any product *j* versus any other product *i* is simply obtained by subtracting both total WTP values (Lusk and Schroeder 2004). Finally, the Wald test (Delta method) was applied to calculate the significance and the confidence intervals for the WTP. We used the NLOGIT 6.0 software and 500 random draws to estimate the coefficients (Chiew 2015).

### Consumers’ environmental attitude

According to Hawcroft and Milfont (2010), environmental attitudes can be observed through psychological tendencies that express positive or negative evaluations of the natural environment and therefore must be inferred. The NEP scale is the most widely used to measure environmental attitudes. It allows general beliefs to be measured based on the relationships between humans and their environment. This scale analyses the relationship that exists between a subject’s beliefs about themselves and nature. It reflects the way in which the human being conceptualises nature, and the way they behave in front of it (Vozmediano and San Juan 2005). It also allows us to identify the predominant latent environmental dimensions of the analysed sample (Gomera et al. 2013). The scale reflects the ways humans conceptualise and interact with ecosystems (Dunlap et al. 2000; Lezak and Thibodeau 2016). In this study, for measuring the consumers´ attitudes towards the environment, the reduced version of the original NEP scale was used, including only the statements related to the ecocentric and anthropocentric attitudes, as mentioned by Orduño Torres et al. (2020). Consumers’ agreement about the NEP scale statements was evaluated according to the 9-point Likert-type scale. After the assessment of the statements of the NEP scale, the principal component analysis (PCA) was performed to identify the dimensionality that characterises the consumers interviewed. The identified dimensions allowed us to define latent factors that were present in the environmental attitudes of the participants (Gomera et al. 2013). According to Durán López et al. (2016), “Ecocentrism recognises the intrinsic value of nature, considers that human beings share the same origin as other species and that the well-being of human communities and biotic communities is complementary”. While “Anthropocentrism views the human being as a being with unique and exceptional characteristics, independent of nature with a higher hierarchical level, can use it, according to his needs and desires without having to be subordinated to his laws”.

### Sustainable consumption

Sustainable consumption behaviour has been widely investigated. In almost all of the empirical researches, this consumer behaviour has been treated as an independent category, but in this research, the sustainable consumption behaviour was analysed through the separate actions that influence behaviour (purchasing, using and recycling habits) (Zeynalova and Namazova 2022; Zhao et al. 2014). Statements used to identify the level of sustainable behaviour involved different actions (as the habit of carrying one’s own bags and using a shopping trolley when going for groceries, buying reusable bags at the store, recycling the food waste, and producing compost from the food waste) and were evaluated according to the 7-point Likert-type scale that indicated the frequency of the action (where 1 = Never, 2 = Rarely, 3 = Sometimes, 4 = Regularly, 5 = Often, 6 = Very often, and 7 = Always). The definition used in this study for food waste was “Food which was originally produced for human consumption but then was discarded or was not consumed by humans, includes food that spoiled prior to disposal and food that was still edible when thrown away” (Kusumowardani et al. 2022; Thyberg and Tonjes 2016). Additionally, the sustainability of the behaviours related to the food waste and recycling was analysed through the use given to the leftovers generated at home.

## Results

The economic and socio-demographic characteristics of the sample showed that 52% corresponded to female consumers. The range of ages was proportionally distributed according to the countries’ populations, corresponding to 13.7%, 20%, 17.6%, 20.2%, and 28.3% to the ranges of (18–24, 25–34, 35–44, 45–54, and 55 or more years old), respectively. About 34% of the consumers stated that they had children under 12 years old in their households, and only 9.6% of consumer households had adults over 70 years old. Close to the majority of interviewed consumers (47.3%) studied secondary education, followed by university studies 43.5%. The highest percentage of consumers with university degrees was in Belgium. The highest percentage of consumers who had an elementary education or had not completed their elementary education was present in the Hungarian case study. In relation to the financial situation of the consumers interviewed, 49% considered it was regular, 26% good, 11% very good, and 15% said that they were in a difficult situation.

### Consumers’ purchase intention

In general terms, the results (Table 4) with regard to consumers´ purchasing intentions towards the three products analysed in each country, showed that conventional products were preferred over products obtained by circular and organic farming systems except in Italy, where circular pork and circular milk were the most preferred. Although circular products showed a low purchase intention rate, there is still a clear potential market for products labelled as being obtained by circular farming systems. Results showed that the purchase intention of circular products represents almost a third of the total rate in the three categories of products (27.24%).

**Table 4:** Aggregated results (%) of purchase intention for each product by country

Products	Pork	Milk	Bread	Pork	Milk	Bread	Pork	Milk	Bread
	Belgium			Croatia			Hungary		
ORG	22.7	22.9	18.1	30.1	24.0	19.1	7.6	5.6	4.9
CONV	40.2	48.2	51.8	34.8	50.6	56.1	55.7	62.9	64.7
CIRC	32.6	25.2	25.7	32.5	21.7	20.4	33.9	21.8	21.7
NONE	4.5	3.7	4.3	2.6	3.7	4.5	2.9	9.7	8.7
	Italy			Poland			Spain		
ORG	27.4	30.7	26.5	34.2	25.6	16.4	24.7	23.5	15.1
CONV	28.8	32.1	35.2	35.0	45.3	57.1	43.7	55.3	56.6
CIRC	40.9	34.9	35.3	27.0	26.0	16.8	29.5	18.8	25.4
NONE	2.9	2.2	2.7	3.8	3.2	9.8	2.0	2.4	2.9

Furthermore, the results showed that the purchase intentions for the three products obtained under the circular farming system (CIRC) were relatively higher than for those products obtained from organic farming (ORG) excluding milk in Spain and Croatia, and pork in Poland, as can be observed in Table 4. Consumers in Italy showed the highest purchase intention levels for the three CIRC products (pork = 40.9, milk = 34.9, and bread = 35.3).

### Consumers’ willingness to pay for circular labelling

The results on preference (Table 5) showed a confidence level of 99%. Thus, the null hypothesis that all coefficients are jointly equal to zero is rejected. The log-likelihood ratio tests were highly significant, showing a significant difference between the preferences of the production farming system and the prices of products for each production farming system. The goodness of fit is assessed through the McFadden’s pseudo-R2, which is highly acceptable, upper than 0.3 (Hensher et al. 2005). The positive/negative sign of the coefficients implies higher/lower levels of utility associated with the products and therefore with their characteristics.

**Table 5:** Purchase intention for each product by country

*β*_s_	Spain	Poland	Italy	Hungary	Croatia	Belgium
RPL pork (500 g each)						
Random *β*_s_						
ASC-ORG*β*_1_	5.64^***^	9.82^***^	6.61^***^	12.72^***^	13.93^***^	7.94^***^
ASC-CONV *β*_2_	6.54^***^	7.72^***^	5.09^***^	10.79^***^	8.55^***^	6.9^***^
ASC-CIRC *β*_3_	6.56^***^	8.8^***^	8.47^***^	24.47^***^	10.14^***^	9.56^***^
Non-random α_s_						
PRICE-ORG*α*_1_	− 0.55^***^	− 0.23^***^	− 0.55^***^	− 0.004^***^	− 0.39^***^	− 0.59^***^
PRICE-CONV α_2_	− 0.84^***^	− 0.15^***^	− 0.54^***^	0.00002	− 0.22^***^	− 0.37^***^
PRICE-CIRC α_3_	− 0.77^***^	− 0.2^***^	− 1.13^***^	− 0.01^***^	− 0.26^***^	− 0.93^***^
S.D of Random *β*_s_						
S.D-ORG	4.68^***^	7.08^***^	4.55^***^	6.62^***^	6.28^***^	5.42^***^
S.D-CONV	2.69^***^	5.19^***^	3.97^***^	6.41^***^	4.14^***^	5.58^***^
S.D-CIRC	3.55^***^	5.49^***^	3.58^***^	6.67^***^	3.82^***^	4.46^***^
Log-likelihood (*θ*)	− 3316.05	− 2992.35	− 2306.30	− 2224.10	− 1442.40	− 2605.28
Pseudo-R^2^	0.40	0.41	0.40	0.54	0.43	0.43
RPL Milk (1 L/pack)						
Random *β*_s_						
ASC-ORG *β*_1_	11.87^***^	8.79^***^	15.49^***^	2.17	3.74^***^	9^***^
ASC-CONV *β*_2_	12.08^***^	10.17^***^	11.15^***^	15.08^***^	7.23^***^	12.12^***^
ASC-CIRC *β*_3_	6.96^***^	9.52^***^	11.14^***^	9.86^***^	5.58^***^	11.17^***^
Non-random α_s_						
PRICE-ORG *α*_1_	− 7.99^***^	− 0.59^***^	− 11.86^***^	− 0.01^***^	− 0.35^*^	− 5.58^***^
PRICE-CONV α_2_	− 6.53^***^	− 1.71^***^	− 9.5^***^	− 0.02^***^	− 0.83^***^	− 9.23^***^
PRICE-CIRC α_3_	1.61	− 1.3^***^	− 7.11^***^	− 0.01^***^	− 0.48^***^	− 8.41^***^
S.D of Random *β*_s_						
S.D-ORG	6.81^***^	5.95^***^	5.28^***^	9.49^***^	6.08^***^	6.94^***^
S.D-CONV	5.81^***^	5.53^***^	4.18^***^	6.08^***^	4.16^***^	5.98^***^
S.D-CIRC	6.11^***^	5.46^***^	4.61^***^	8.11^***^	4^***^	5.6^***^
Log-likelihood (θ)	− 2727.02	− 3094.00	− 2073.35	− 2059.27	− 1435.86	− 2341.72
Pseudo-*R*^2^	0.46	0.41	0.40	0.54	0.45	0.48
RPL Bread (450 g each)						
Random *β*_s_						
ASC-ORG *β*_1_	4.36^***^	4.46^***^	8.37^***^	1.3	5.01^***^	9.53^***^
ASC-CONV *β*_2_	9.65^***^	10.28^***^	7.6^***^	14.79^***^	9.61^***^	12.66^***^
ASC-CIRC *β*_3_	7.13^***^	8.38^***^	9.77^***^	13.51^***^	7.83^***^	12.89^***^
Non-random α_s_						
PRICE-ORG *α*_1_	− 1.43^***^	− 0.36^***^	− 3.61^***^	− 0.01^*^	− 0.31^**^	− 4.33^***^
PRICE-CONV α_2_	− 5.09^***^	− 0.97^***^	− 3.12^**^	− 0.01^***^	− 0.55^***^	− 4.57^***^
PRICE-CIRC α_3_	− 2.81^***^	− 0.74^***^	− 5.75^***^	− 0.02^***^	− 0.41^***^	− 6.28^***^
S.D of Random *β*_s_						
S.D-ORG	5.96^***^	11.03^***^	5.16^***^	11.31^***^	7.75^***^	8.37^***^
S.D-CONV	3.82^***^	5.42^***^	3.98^***^	7.38^***^	4.59^***^	6.11^***^
S.D-CIRC	5.27^***^	7.11^***^	4.89^***^	9.66^***^	5.2^***^	7.03^***^
Log-likelihood (*θ*)	− 2499.00	− 2718.75	− 1819.10	− 1671.76	− 936.33	− 2113.16
Pseudo-R^2^	0.47	0.44	0.39	0.57	0.46	0.51

^***^, ^**^, and ^*^ = = > significance at 1, 5, and 10 level

In this context, the estimated models showed that all coefficients are statistically significant in all countries and between production farming systems. However, estimates cannot be compared between countries due to the scale parameter, and comparisons should be evaluated only at the WTP levels. It is important to remember that price coefficients were considered fixed (i.e. non-random) to ensure that estimated total WTP were normally distributed and with a finite moment.

Results of the model for each product showed that all significative prices present negative sing, which indicates that there was an inverse relationship between price and the probability of their election, confirming the sensitivity to the price levels when selecting their preferred products from simulated purchase situations, and highlighting a decreasing demand trend when prices increase as expected in the majority of the cases. Additionally, all estimated standard deviations of the random coefficients (ASCs) were highly statistically significant, confirming the presence of observed heterogeneity around the mean (i.e. utilities of the products farming system) and thus the suitability of used model specification. The results also showed a high level of heterogeneity of marginal utilities of the ASC (which represents the marginal utility of a product relative to the NONE option) for circular farming products compared to the ASC of organic and conventional. Focusing on the pork product from circular farming, the results showed that it received the highest preference (marginal utility) in Italy, Belgium, and Hungary, while in Spain, Croatia, and Poland, marginal utility was higher than conventional pork, but relatively lower than the organic one. The milk results also showed that the utility of milk from circular farming was relatively higher than the organic one in Hungary, Croatia, and Belgium. However, in Spain, the utilities associated with this milk from circular farming received the lowest utility level. For the bread, the results showed the higher marginal utility of bread from circular farming compared to organic farming. However, this utility level was lower than the utility obtained from conventional bread.

In all cases, consumers’ WTP was calculated including the marginal utility of the price for the preference analysis. The WTP results are in (Table 6) for each product category. For the pork product, the expected WTP showed positive and significant values for the products of circular farming in all countries. This means that all the consumers interviewed are willing to pay an additional premium for circular pork. The WTP for pork from circular farming was higher than the conventional one in Spain (9%), Hungary (100%—WTP for conventional pork was non-significant), and Croatia (2%), and also, the WTP for circular pork was higher than for organic in Poland (6%) and Croatia (9%). In Poland and Croatia, although the organic pork received the highest utility, consumers were not willing to pay too much for it. These results could also be associated with the high price level presented. In Belgium, the highest utility was obtained for pork produced by circular farming (Pork ASC-CIRC *β*_**3**_ = 9.56). However, the WTP for circular pork (10.25) was relatively low, compared to the pork produced in an organic farming system (13.44). This outcome could also be related to the lower price level presented for conventional pork which may have influenced the results.

**Table 6:** WTP for pork, milk, and bread products by country

WTP	Spain	Poland	Italy	Hungary	Croatia	Belgium
*Pork (500 g each)*						
WTP-ORG *β*_1_/*α*_1_	10.28^***^	9.19^***^	12.04^***^	8.49^***^	7.86^***^	13.44^***^
(95) confidence interval	(7.40–13.15)	(6.47–11.91)	(9.36–14.72)	(6.36–10.61)	(6.55–5.61)	(10.80–16.07)
WTP-CONV *β*_2_/*α*_2_	7.75^***^	11.53^***^	9.36^***^	− 12.64	8.42^***^	18.41^***^
(95) confidence interval	(6.09–9.41)	(3.36–19.7)	(4.55–4.162)	(− 8.22–24.1)	(5.92–6.69)	(8.55–28.27)
WTP-CIR *β*_3_/*α*_3_	8.46^***^	9.73^***^	7.52^***^	5.84^***^	8.6^***^	10.25^***^
(95) confidence interval	(6.60–10.32)	(5.12–14.34)	(6.04–9.01)	(5.04–6.64)	(6.61–6.5)	(8.27–12.21)
WTP	Spain	Poland	Italy	Hungary	Croatia	Belgium
*Milk (1 L/pack)*						
WTP-ORG *β*_1_/*α*_1_	1.49^***^	3.23^***^	1.31^***^	0.55	1.43^***^	1.61^***^
(95) confidence interval	(0.93–2.04)	(2.51–3.94)	(1.11–1.49)	(− 0.18–1.27)	(0.73–2.14)	(1.06–2.16)
WTP-CONV *β*_2_/*α*_2_	1.85^***^	1.29^***^	1.17^***^	1.71^***^	1.16^***^	1.31^***^
(95) confidence interval	(1.04–2.65)	(0.83–1.75)	(0.90–1.43)	(1.53–1.89)	(0.86–1.47)	(1.01–1.62)
WTP-CIR *β*_3_/*α*_3_	− 4.33	1.58^***^	1.57^***^	1.83^***^	1.55^***^	1.33^***^
(95) confidence interval	(− 7.60–1.03)	(0.92–0.08)	(1.15–1.97)	(1.5–2.17)	(0.92–2.19)	(1.00–1.65)
WTP	Spain	Poland	Italy	Hungary	Croatia	Belgium
*Bread (450 g each)*						
WTP-ORG *β*_1_/*α*_1_	3.04^***^	2.73^***^	2.32^***^	0.5	2.19^***^	2.20^***^
(95) confidence interval	(1.90–4.17)	(1.57–3.88)	(1.52–3.11)	(− 1.45–2.45)	(0.98–3.39)	(1.50–2.89)
WTP-CONV *β*_2_/*α*_2_	1.90^***^	2.30^***^	2.43^***^	2.97^***^	2.34^***^	2.77^***^
(95) confidence interval	(1.50–2.28)	(1.93–2.66)	(0.69–4.17)	(2.28–3.65)	(1.46–3.21)	(1.54–3.99)
WTP-CIR *β*_3_/*α*_3_	2.54^***^	2.46^***^	1.70^***^	2.21^***^	2.53^***^	2.05^***^
(95) confidence interval	(1.65–3.41)	(1.94–2.98)	(1.10–2.29)	(1.73–2.70)	(1.36–3.69)	(1.42–2.68)

^***^, ^**^, and ^*^ = = > significance at 1, 5, and 10 level

The estimated results for milk products showed positive and significant WTP for the milk obtained from the circular farming system, except in Spain where results showed a non-significant WTP value. The WTP for milk produced under a circular farming system in comparison with the conventional was 20% higher in Poland, 34% in Italy and Croatia, 7% in Hungary, and 2% in Belgium. Hungary and Croatia had the highest expected WTP for the circular milk, while in Poland and Belgium, the highest WTP was for organic milk. In the case of bread, the results showed that the estimated WTP was significant in all countries and production systems, with the exception of the estimated WTP for organic bread, which was not significant in Hungary, nor was the utility that it represented for Hungarian consumers. They obtained the highest utility from conventional bread. The results also showed that bread produced from circular farming received a greater WTP than conventional bread in Spain, Poland, and Croatia (34%, 7%, and 8% more than conventional bread, respectively). The estimated WTP for circular bread was greater (16%) than the WTP for organic bread for Croatian consumers. Although circular bread represents the highest marginal utility for Italian and Belgian consumers, the results show that they are not willing to pay a premium price for this product compared to conventional products.

### Consumers’ environmental attitude

The consumers´ environmental attitude, analysed with the reduced NEP scale, allowed us to identify two latent dimensions. The first dimension was related to ecocentric attitude and reflects pro-environmental behaviour, seeking to achieve a balance between the human being and the natural ecosystem, while the second was related to an anthropocentric attitude and reflects an attitude towards carrying out actions that satisfy needs and achieve human well-being above everything else (living beings, the environment, valuing the environment for its benefits to people, etc.). Respondents who give more importance to the anthropocentric dimension conceive the human being and its interests as the centre of everything. Considering the superiority of each latent dimension of the NEP scale, respondents in each country were classified according to their ecocentric and anthropocentric attitudes (Table 7). The results showed that consumers’ environmental attitudes were heterogeneous at the country level. The results also showed that the ecocentric attitude was more pronounced in Croatia, Hungary, and Italy, while the anthropocentric attitude was more prominent in Belgium, Poland, and Spain. At the global level, the anthropocentric attitude was predominant (52.3%).

**Table 7:** Consumers’ environmental attitude by country (%)

Attitude	Belgium	Croatia	Hungary	Italy	Poland	Spain	Global attitude
Ecocentric	43.47	53.16	50.91	51.28	45.38	45.52	47.67
Anthropocentric	56.53	46.84	49.09	48.72	54.62	54.48	52.33

### Consumption behaviour (purchasing, food waste, and recycling habits)

The results obtained in the analysis of consumption behaviours through purchasing, food waste, and recycling habits, showed that the percentage of consumers who compost food waste was higher in Belgium, Croatia, and Hungary compared to Italy, Spain, and Poland. More than half of the consumers interviewed (3177 respondents) usually generate leftovers from meals they prepare at home. The highest percentage was found in Belgium and the lowest in Poland. However, only a small share of leftovers was thrown into the garbage (wasted), with a percentage ranging from 6.8% in Italy to 16.4% in Croatia.

### Factors affecting consumers’ preferences

As mentioned before, hybrid RPL models were estimated (RPL + interactions). The results for each product (pork, milk, and bread) and country, including environmental attitudes, consumption behaviours, and consumers´ economic and socio-demographic characteristics, are shown in Tables 8, 9, and 10. Their inclusion generated better goodness of fit model indicators (AIC and log-likelihood—smallest, and R-squared—biggest) in almost all the models.

**Table 8:** Pork purchase intention by country

			Spain	Poland	Italy	Hungary	Croatia	Belgium
	PORK	Random parameters						
		ORG	4.011^***^	3.956^***^	1.568	5.133^**^	12.147^***^	4.716^***^
		CONV	6.901^***^	3.422^***^	4.709^***^	7.264^***^	7.929^***^	5.737^***^
		CIRC	5.816^***^	4.585^***^	4.878^***^	20.123^***^	8.192^***^	6.913^***^
		Non-random parameters in utility functions						
		PREU_ORG	− 0.541^***^	− 0.241^***^	− 0.537^***^	− 0.008^***^	− 0.388^***^	− 0.601^***^
		PREU_CON	− 0.843^***^	− 0.148^***^	− 0.522^***^	0.000	− 0.228^***^	− 0.404^***^
		PREU_CRC	− 0.815^***^	− 0.216^***^	− 1.141^***^	− 0.025^***^	− 0.271^***^	− 1.009^***^
Attitudinal and behavioural factors	Ecocentric environmental attitude	ORG_ECO	0.409^**^	0.442^*^			0.981^**^	0.867^***^
		CONV_ECO	− 0.396^**^		− 0.589^**^		0.635^**^	
		CIRC_ECO	0.469^***^	0.755^***^		1.414^***^	0.770^***^	1.126^***^
	Anthropocentric environmental attitude	ORG_ANT						
		CONV_ANT	0.621^***^	0.576^**^	0.876^***^	0.638^**^		1.001^***^
		CIRC_ANT						
	Sustainable purchasing behaviour	ORG_SP	0.411^**^	0.487^**^	0.750^***^			0.480^***^
		CONV_SP		0.293^*^				− 0.307^*^
		CIRC_SP						−
	Food waste and recycling behaviour (use of leftovers)	ORG_SL						− 0.232^**^
		CONV_SL			0.189^*^			
		CIRC_SL			0.341^***^			
Economic and socio-demographic characteristics	Female	ORG_GEN						
		CON_GEN		− 1.374^***^	− 1.710^***^	− 1.074^*^	− 1.165^**^	
		CIRC_GEN			− 1.171^***^	− 1.651^***^		
	Age > 54	ORG_AGE	− 1.121^***^	− 1.113^*^				
		CON_AGE				1.877^***^		
		CIRC_AGE	− 0.833^**^	− 0.969^**^		1.564^***^		
	Not completed OR elementary education	ORG_EDU	− 0.804^*^		− 1.658^**^			
		CON_EDU				1.261^*^		1.386^*^
		CIR_EDU		− 0.643^*^	− 2.611^***^	− 1.785^***^		–
	Financial situation good/very good	ORG_FNB		1.630^***^	0.965^*^	1.434^*^		1.062^**^
		CON_FNB						1.342^***^
		CIRC_FNB						
		Std. Devs.						
		NsORG	3.584^***^		3.500^***^	4.803^***^	5.684^***^	4.194^***^
		NsCONV	2.669^***^		−	3.674^***^		4.376^***^
		NsCIRC	2.038^***^		2.468^***^	3.619^***^	2.578^***^	2.935^***^
		Goodness of fit						
	Log-likelihood		− 3262.95	− 2998.87	− 2247.28	− 2171.29	− 1439.22	− 2574.62
	McFadden pseudo-R-squared		0.41	0.41	0.42	0.55	0.43	0.44
	AIC		6591.90	6063.70	4560.60	4408.60	2944.40	5215.20

^***^, ^**^, and ^*^ = = > significance at 1%, 5%, and 1 level

**Table 9:** Milk purchase intention by country

			Spain	Poland	Italy	Hungary	Croatia	Belgium
	Milk	Random parameters						
		ORG	6.218^***^	3.813^***^	9.128^***^	0.911	3.474^*^	5.607^***^
		CONV	8.184^***^	5.765^***^	6.838^***^	12.226^***^	8.171^***^	9.321^***^
		CIRC	1.877^**^	4.372^***^	5.733^***^	6.371^***^	5.053^***^	8.017^***^
		Non-random parameters in utility functions						
		PREU_ORG	− 7.684^***^	− 0.590^***^	− 12.205^***^	− 0.025^***^	− 0.345^*^	− 5.731^***^
		PREU_CON	− 5.873^***^	− 1.832^***^	− 9.805^***^	− 0.052^***^	− 0.815^***^	− 9.473^***^
		PREU_CRC	1.489^***^	− 1.410^***^	− 7.463^***^	− 0.033^***^	− 0.548^***^	− 9.171^***^
Attitudinal and behavioural factors	Ecocentric environmental attitude	ORG_ECO	0.993^***^	0.691^***^				1.355^***^
		CONV_ECO		0.379^*^			0.574^*^	
		CIRC_ECO	0.832^***^	0.708^***^	0.469^**^	1.143^***^		1.743^***^
	Anthropocentric environmental attitude	ORG_ANT						
		CONV_ANT	0.675^***^	0.431^*^	0.754^***^	0.584^**^		
		CIRC_ANT						
	Sustainable purchasing behaviour	ORG_SP		0.350^**^	0.895^***^			
		CONV_SP			0.390^**^		− 0.524^**^	− 0.322^*^
		CIRC_SP					− 0.418^**^	
	Food waste and recycling behaviour (use of leftovers)	ORG_SL						
		CONV_SL	− 0.291^***^	− 0.196^**^		− 0.223^**^		
		CIRC_SL				− 0.229^*^		
Economic and socio-demographic characteristics	Female	ORG_GEN			− 1.049^**^	− 2.401^***^		
		CON_GEN		0.745^*^		− 0.840^**^	1.126^*^	
		CIRC_GEN	0.620^*^			− 1.075^**^	1.370^**^	1.151^**^
	Age > 54	ORG_AGE					− 1.801^**^	
		CON_AGE	1.584^***^			0.819^*^	− 1.171^*^	2.030^***^
		CIRC_AGE				0.907^*^		
	Not completed OR elementary education	ORG_EDU			− 2.823^**^	2.126^**^		
		CON_EDU						
		CIR_EDU	− 1.086^**^					
	Financial situation good/very good	ORG_FNB						
		CON_FNB			− 0.844^*^	0.917^*^		
		CIRC_FNB				1.497^***^		
		Std. Devs.						
		NsORG	3.853^***^		4.034^***^	6.393^***^	4.452^***^	4.673^***^
		NsCONV	3.490^***^			4.137^***^		4.592^***^
		NsCIRC	1.965^***^		2.367^***^	4.515^***^	2.538^***^	3.078^***^
		Goodness of fit						
	Log-likelihood		− 2737.05	− 3130.81	− 2039.80	− 2078.25	− 1442.98	− 2294.90
	McFadden pseudo-R-squared		0.46	0.41	0.41	0.53	0.45	0.49
	AIC		5540.10	6327.60	4145.60	4222.50	2952.00	4655.80

^***^, ^**^, and ^*^ = = > significance at 1%, 5%, and 1 level

**Table 10:** Bread purchase intention by country

			Spain	Poland	Italy	Hungary	Croatia	Belgium
	Bread	Random parameters						
		ORG	0.512	− 0.701	2.805^**^	− 5.428^*^	6.151^***^	7.607^***^
		CONV	8.353^***^	6.320^***^	2.733^**^	10.718^***^	9.783^***^	9.827^***^
		CIRC	4.834^***^	3.758^***^	4.985^***^	8.358^***^	5.614^***^	8.824^***^
		Non-random parameters						
		PREU_ORG	− 1.493^***^	− 0.366^***^	3.583^***^	− 0.011	− 0.318^***^	− 4.375^***^
		PREU_CON	− 5.062^***^	− 0.992^***^	− 3.096^**^	− 0.030^***^	− 0.564^***^	− 4.558^***^
		PREU_CRC	− 3.051^***^	− 0.767^***^	− 6.034^***^	− 0.039^***^	− 0.457^***^	− 6.761^***^
Attitudinal and behavioural factors	Ecocentric environmental attitude	ORG_ECO	0.474^*^					1.904^***^
		CONV_ECO						
		CIRC_ECO	0.995^***^		1.033^***^	1.308^***^		1.735^***^
	Anthropocentric environmental attitude	ORG_ANT				− 0.946^**^		
		CONV_ANT	0.663^***^		0.836^***^	0.724^**^		1.049^***^
		CIRC_ANT		− 0.374^*^			− 0.970^***^	
	Sustainable purchasing behaviour	ORG_SP	0.633^***^	0.544^*^	0.577^**^	0.884^**^		
		CONV_SP					− 0.550^**^	
		CIRC_SP						
	Food waste and recycling behaviour (use of leftovers)	ORG_SL				− 0.376^*^	− 0.379^*^	− 0.361^**^
		CONV_SL		− 0.107^*^	0.218^*^		− 0.292^*^	− 0.303^**^
		CIRC_SL			0.358^***^			
Economic and socio-demographic characteristics	Female	ORG_GEN	− 0.971^*^		− 1.149^**^		− 1.703^*^	− 1.301^*^
		CON_GEN						
		CIRC_GEN						
	Age > 54	ORG_AGE	− 1.453^**^					
		CON_AGE						1.657^**^
		CIRC_AGE				1.416^**^		
	Not completed OR elementary education	ORG_EDU			− 3.479^***^	− 2.305^*^		
		CON_EDU						− 1.568^*^
		CIR_EDU	− 1.608^***^		− 3.766^***^	− 1.645^**^		− 1.816^**^
	Financial situation good/very good	ORG_FNB	1.215^**^			2.300^**^		
		CON_FNB			− 1.335^**^			
		CIRC_FNB				1.477^**^		
		Std. Devs.						
		NsORG	4.393^***^		3.261^***^	6.993^***^	5.473^***^	5.816^***^
		NsCONV	2.822^***^			4.601^***^		4.557^***^
		NsCIRC	2.520^***^		2.525^***^	4.856^***^	2.750^***^	3.809^***^
		Goodness of fit						
	Log-likelihood		− 2461.30	− 2844.12	− 1779.61	− 1699.25	− 933.53	− 2078.47
	McFadden pseudo-R-squared		0.48	0.42	0.41	0.56	0.46	0.51
	AIC		4988.60	5754.20	3625.20	3464.50	1933.10	4222.90

^***^, ^**^, and ^*^ = = > significance at 1%, 5%, and 1 level

In the case of pork meat models, the results showed that consumers´ ecocentric attitude was related to an increased preference for circular pork, while the anthropocentric attitude did not have any effect on preferences towards circular pork, nor did sustainable purchasing behaviour have effect on the preference for circular pork. Sustainable behaviour regarding food waste and recycling only had a significant effect on the preference for circular pork among Italian consumers. Women had a negative effect on preference of pork meat obtained by a circular system in Italy and Hungary. Being more than 54 years old had a negative effect on the preference for circular pork in Spain and Poland, while in Hungary, this same characteristic affected this preference positively. Regarding consumers with elementary education or an incomplete elementary education, the results showed that the preference for circular pork was negatively affected in Poland, Italy, and Hungary. A good financial situation had no effect on the preference for pork obtained by a circular system.

For milk, the estimated models showed that an ecocentric attitude was principally related to a positive effect on consumers´ preference towards milk obtained by the circular system, with the exception of Croatian consumers. Anthropocentric attitude, sustainable purchasing behaviour, and sustainable behaviours regarding food waste and recycling did not have an effect on the preference for milk obtained by a circular farming system. In Spain, Croatia, and Belgium, women influenced positively the preference for circular milk, while in Hungary, the effect was negative. In Hungary, if consumers were over 54 years old or had a good financial situation, this had a positive effect on the preference for circular milk. Regarding education, Spanish consumers having only an elementary education or less had a negative effect on the preference for circular milk.

Through the analyses of bread preferences, the results indicated that an ecocentric attitude had a positive effect on circular bread´ preference in almost all countries, except for Polish and Croatian consumers, while in these two countries, the anthropocentric attitude affected negatively the preference for circular bread. Sustainable behaviour towards food waste and recycling had a positive effect on the circular bread preference among Italian consumers. Gender had no effect on the preferences for bread obtained by the different production systems. With respect to age, being more than 54 years old increased the preference for circular bread in Hungary. With regard to the education level, consumers having an elementary education or an incomplete elementary education had a negative effect on the preference for circular bread, with the exception of consumers from Poland and Croatia. Finally, bread obtained by a circular system was positively affected by a good financial situation in Hungary.

## Discussion

The factors affecting consumers’ preferences showed that consumers´ ecocentric attitudes play a positive role, affecting the preference for the circular farming system. Conversely, anthropocentric attitudes increase the preference for less sustainable food products, supporting hypothesis “*H1: Ecocentric environmental attitudes are positively related to a preference for circular food*” and “*H2: Anthropocentric environmental attitudes are negatively related to a preference for circular food*”. These results underscore the fact that environmental attitudes are pertinent factors that influence consumers’ willingness to pay (WTP) for circular products, as suggested by the TPB and VBN theories. According to the TPB, ecocentric attitudes indicate a favourable disposition towards purchasing sustainable products, which is indicative of consumers’ conviction regarding the advantages of such decisions. The VBN theory posits that the ecocentric values of individuals promote stronger pro-environmental beliefs and norms, which, in turn, drive their preference for circular farming systems. This is consistent with the assumption that environmental attitudes greatly influence WTP for circular products. These results are in line with the findings obtained in the study by Rahman et al. (2014), who showed that consumers with high ecocentric attitude assigned a higher preference rating to more sustainable products than anthropocentric participants. Similarly, Mustapa and Kallas (2023) argued that consumers with an ecocentric attitude have more willingness to purchase food obtained through sustainable practices. According to Zsóka et al. (2013), although environmental attitudes are related to the perception of more sustainable consumption need, they are not always reflected in daily activities. Young et al. (2010) also concluded that those consumers who were concerned about the environment could be incentivised by single labels helping them to concentrate their efforts on purchasing decisions.

The results regarding to consumption behaviours indicated that consumers who follow sustainable purchasing habits preferred organic products, due to their perception of their sustainability. Lazaroiu et al. (2019) argued that consumers with sustainable purchasing behaviour are willing to pay premium for environmentally sustainable products, similarly to those proposed in our study and highlight the importance of labels to inform consumer in their decision-making. However, non-significant results were identified for sustainable purchasing behaviours and willingness to pay for food obtained from the proposed circular production, thereby rejecting the third hypothesis *“H3: Sustainable food purchasing habits are positively related to a preference for food labelled as circular*”. This result may be attributable to the absence of a clear understanding of the characteristics and advantages of circular production systems provided to consumer in the choice cards, in contrast with organic foods, which are more familiar to consumers. This result indicates the need of more extensive dissemination of information regarding production in the context of circularity initiatives. Enhanced educational efforts and the development of standardised labelling could help bridge this gap. Consumers currently recognise and trust organic labels, which effectively communicate the immediate health benefits of organic products. In contrast, the benefits of circular production, such as reduced GHG emissions and nutrient recovery, are less tangible and more abstract. A strategic communication strategy is crucial to convey the environmental and personal advantages of circular food production, consistent with the conclusions of Young et al. (2010) about the influence of clearly sustainable food labelling on consumer purchase behaviour.

Consumers who have sustainable behaviours, related to low food waste generation and food recycling, preferred less the conventional food in all countries, in the same way of hypothesis “*H4: Sustainable food waste management and recycling habits are positively related to a preference for food labelled as circular”.* The hypothesis is additionally corroborated in the context of Italy. A substantial increase in the preference for pork meat and milk obtained through the circular production system was observed among Italian consumers who prioritise food waste management and recycling. This suggests that their sustainable consumption behaviours are more closely aligned with circular food products, which may be attributed to the increased awareness and acceptance of these practices in Italy. The preferences observed may be influenced by varying levels of awareness and education about the benefits of circular production systems. In countries like Italy, where sustainable practices and circular economy concepts are more widely promoted (Ghisellini and Ulgiati 2020), consumers are more likely to understand and better perceive the advantages of circular food production, thus influencing their preferences. This suggests a need to enhance knowledge campaigns about the benefits of circular food systems. Clear labelling and certification for circular products can also help bridge the information gap. However, it is important to note that the impact of recycling behaviours on purchasing decisions is not universally agreed upon. For instance, Jimenez et al. (2014) argued that recycling had no effect on the purchase of organic products. This suggests that while sustainable behaviours like recycling may influence some aspects of consumption, their effect can vary depending on the type of product, and the specific environmental practices consumers are considering.

Regarding the socioeconomic consumer characteristics, gender and age had high heterogeneity effect on preferences towards circular food products according to the product and the country. Financial conditions positively influenced Hungarian consumers’ choices for circular food. The hypothesis* “H5.1:* Younger individuals exhibit a higher willingness to pay (*WTP) for circular food than older consumers”,* was rejected, indicating that younger consumers were with the preferences for food produced in a circular system. These results are contrary to those obtained by Ali et al. (2021) and Jimenez et al. (2014). In terms of gender, the results demonstrated that being female has no influence on the choice for circular food, contrary to our hypothesis build on Li and Kallas (2021) “*H5.2: Women are likely to pay more for circular food products”*. With respect to “*H5.3: A good financial situation is positively related to a willingness to pay more for circular food products*”, only Hungary’s results showed a positive relationship with the preference for circular milk and circular bread. Similar results were found by Villanueva (2016), which suggests that the level of income is one of the factors behind the purchase of more sustainable food supporting the environment.

Regarding the hypothesis “*H6: The WTP in percentage terms is higher for less expensive products, such as milk or bread, than for meat*”, the comparison of the estimated WTP with the average price of each product based on the production system utilised, revealed that, despite the price of pork meat (500 gr) being greater than that of milk (1 L) and bread (450 gr), the WTP for pork in percentage terms exceeded than for milk or bread, contrary to the proposed hypothesis. Results previously described demostrated that the preferences for food products obtained by different production systems varied depending on the country and type of product analysed. This demostrates the indirect impact of socioeconomic factors on sustainable food choices which confirm the hypothesis *H7: Preferences are related to country context*. Results showed that sustainable behaviours regarding food waste and recycling in Italy have a positive impact on preferences for food obtained through a circular production system, while a low level of education has a negative impact. This latter result is consistent across all countries, particularly in the case of circular pork and bread. In Hungary, having good income and being over the age of 54 were the criteria that positively influenced circular food preferences. In Belgium, the ecocentric environmental attitude positively affected the preference of the three circular products, whereas in Spain, the favourable effect was only for circular bread and milk, and in Croatia only for circular pork.

All of these patterns emphasise that circularity is a complex topic with multidimensional characteristics shaped by a wide range of interrelated influences. Each country’s unique condition, policies, and public awareness campaigns related to sustainability can impact consumer preferences. In essence, these disparities are likely the result of a combination of national contexts, financial factors, cultural values, and education. The results aligned with those obtained by Falk et al. (2018). In their conclusion, they argue that the wide range of preferences between and within countries is linked to individual and aggregated characteristics.

One significant empirical drawbacks of this study is its reliance on hypothetical purchasing scenarios within the experimental economic framework. This method may introduce potential errors stemming from hypothetical bias, as participants do not encounter actual purchasing scenarios. As a result, the findings may not precisely represent actual behaviour in the real world. A further limitation is the absence of comparability of results across countries in monetary terms, as each nation possesses its own price vector for each examined food product. These limitations highlight the need for careful analysis when interpreting results and undertaking cross-country comparisons. Furthermore, while we chose food products from various categories, we excluded other food items closely associated with circular production systems, such as vegetables. Broadening the ranfe of food products examined could yield a more thorough comprehension of consumer preferences in circular production systems.

## Conclusions

The demand for more sustainable food production systems and consumption is becoming fundamental to sustainable development. There is increased interest in analysing consumers’ purchase intentions and WTP for food obtained by more sustainable production systems, such as circular farming. Circular farming focuses on using minimal amounts of external inputs, closing nutrient loops, regenerating soils, and minimising impact on the environment. Understanding the WTP for sustainable products allows policymakers and multi-agent stakeholders to carry out and design more socially acceptable policy actions that ensure sustainable food production.

The results showed that pork, milk, and bread products obtained under circular farming systems received relatively low rates of purchase intention compared to conventional products. However, the results obtained in this study showed that there is a clear potential market for products as pork, milk, and bread, labelled as obtained under a circular farming system. The global average rate of purchase intention for the three products categories was 27.24% as a potential market share. These results confirm the substitutability characteristics across the products from the different production systems in a potential marketplace, and highlight the potential acceptance of products from circular farming. Additionally, the results highlighted the importance of consumers’ environmental attitudes and their socioeconomic characteristics in determining their preferences for environmentally sustainable products. The results also highlighted how in all countries, consumers’ desire to see farmers more committed to environmental protection by adopting innovations in the production of renewable bioenergy and recycling organic waste.

All of the previous results allow us to suggest to governments, policymakers, and other sectors involved in sustainable food production, standardised labels for food obtained by circular farming systems, the design of educational programmes to increase knowledge about the problems generated by unsustainable consumption habits and the promotion of circular product consumption as a sustainable consumer behaviour, helping consumers internalise appropriate messages about reuse, the benefits of recycling for the environment and society, and altering their preferences. Experts should provide consumers with detailed information about the importance of environmentally friendly products to help consumers make the correct choices. Finally, the results obtained in this study derive from a period before the worldwide increase in inflation. Furthermore, this study depends on the hypothetical nature of WTP elicitation inherent to consumers´ stated preferences valuation methodology, rather than on revealed preferences.

## Supplementary Information

Additional file1 (DOCX 60 KB)

## Data Availability

The dataset generated and analysed during the current study is available in Zenodo a public repository [https://doi.org/10.5281/zenodo. 8338014].
