# Supplementary material for: The role of environmental attitudes and consumption patterns in consumers’ preferences for sustainable food from circular farming system: a six EU case studies
Source: Agric Food Econ. 2025 Feb 19;13(1):7. doi: 10.1186/s40100-025-00350-0 (PMC11839691; doi:10.1186/s40100-025-00350-0)
Supplement: Supplementary file 1 — Additional file1 (DOCX 60 KB) [file 40100_2025_350_MOESM1_ESM.docx]

**The role of environmental attitudes and consumption patterns in Consumers' preferences for sustainable food from circular farming system: A six EU case studies**

**Appendix 1**

Descriptions of the conventional, organic, and circular production systems used to obtain the specific selected products in the labelled choice experiment.

1. For Pork and milk

**Conventional Livestock**: Livestock is typically housed under constantly controlled temperature, light, and humidity control. Livestock are primarily fed weed feed and fodder. It uses high-performance meat breeds that are tailored to market demand and produces homogeneous products (cut, size, and volume) that meet large-scale marketing requirements. It is subject to a general livestock regulation that governs its operations in terms of food, hygiene, and production, as well as the prohibition on the use of growth hormones. Antibiotic use on livestock farms is closely monitored and supervised. The use of drugs for disease control must be authorised and administered through veterinary prescription while adhering to the principles of good veterinary practice (Chander et al., 2011; Sundrum, 2001; Slagboom et al., 2016; van Wagenberg et al., 2017).

**Organic Livestock**: Livestock is raised under strict guidelines for living conditions, medical treatment, and animal welfare. Livestock are fed grass, fodder, or certified organic feed. GMO feed cannot be used, and antibiotics can only be administered to animals in exceptional circumstances. However, the products (milk, meat) require a longer quarantine period following treatment. Animals must have permanent access to the outdoors, and the space should have a low animal density. The regulations emphasise improving animal welfare throughout their lifespan, controlling their transport and slaughter conditions (Slagboom et al., 2016; Nowak et al., 2013; Läpple et al., 2013).

**Circular Livestock**: Animal husbandry produces not only meat, milk, and eggs but also manure, urine, heat, ammonia, methane, and CO2, all of which, if not controlled, can have a negative impact on the environment. These materials are frequently underutilised and, according to some farmers, are considered waste. In contrast, circular farming raises livestock in conventional farming conditions while incorporating circular economy principles. As a result, these farming systems incorporate a variety of technologies, solutions, and farming practices to improve the recycling of carbon, nitrogen, phosphorus, energy, and water by emphasising more efficient nutrient use, improving animal feeding, reducing residues and emissions, and recovering and reusing nutrients from biowaste. Pig slurry and manure are treated using a variety of techniques to produce bioenergy (biogas) and bio-based fertilisers. In the case of cattle farming, the dairy farm uses wastewater to produce algae as a new source of proteins (animal feeding), and the milk industry uses dairy processing residues to produce fertiliser and improve soil fertility (Yu-nan Xue et al., 2019; Koppelmäki et al., 2021; Qiong Yue et al., 2022; Rodias et al., 2020; Vlachokostas et al., 2021)***.***

1. For Bread

**Conventional Farming**. Bread-quality wheat flour is made from improved varieties obtained through traditional breeding methods, excluding biotechnology (GMO). These varieties have higher yields, better pest and disease resistance, and larger seeds. It is a type of variety that is adapted to market demand and produces a homogeneous crop (e.g., grain protein content) and flour quality (e.g., bread volume index) to meet large-scale marketing requirements. The use of synthetic pesticides and chemical fertilisers is allowed (Nowak et al., 2013).

**Organic Farming:** Organic bread wheat cultivation requires the use of specific certified organic practices and production guidelines. Genetically modified organisms (GMOs) and seed radiation (to eradicate seed-borne diseases and pests) are strictly prohibited. Synthetic phytosanitary treatments (pesticides) and chemical fertilisers are prohibited or severely restricted; however, the use of organic fertilisers (e.g., manures) approved for organic farming is permitted, albeit within certain application limits. Preventive methods for insect and disease control are allowed, including crop rotation, the use of resistant and genetically improved varieties, with traditional breeding methods. (Puech et al., 2014; Nowak et al., 2013).

**Circular Farming**: Bread-wheat production here involves using agricultural biomass (e.g., animal manure, crop residues), processing waste, and food industry by products as renewable resources. This farming system is considered multifunctional because it produces both food, feed, and various organic residues that can be used as fodder for livestock, bio-based fertilisers, or a source of bioenergy. (Tagarakis et al., 2021). In the case of bread production, cereals for flour are grown using crop management systems that improve soil fertility and organic matter content through crop rotations, cover crops, and no-tillage practices, and the crops are fertilised with recycled, bio-based fertilisers.

Table 1: Descriptive results of the samples (% of all responses)

|  | Country | Spain | Poland | Italy | Hungary | Croatia | Belgium | Global |
| --- | --- | --- | --- | --- | --- | --- | --- | --- |
|  | Sample Size | 1050 | 1040 | 780 | 988 | 506 | 998 | 5362 |
| Gender | Male | 49.5 | 40.5 | 47.1 | 48.8 | 54.7 | 52.0 | 48.2 |
|  | Female | 50.5 | 59.5 | 52.9 | 51.2 | 45.3 | 48.0 | 51.8 |
| Range Age | 18-24 years | 9.4 | 21.4 | 11.4 | 10.0 | 21.3 | 11.7 | 13.7 |
|  | 25-34 years | 15.6 | 24.6 | 22.1 | 15.4 | 28.3 | 19.1 | 20.1 |
|  | 35-44 years | 21.5 | 13.2 | 17.4 | 21.6 | 8.5 | 19.1 | 17.6 |
|  | 45-54 years | 21.8 | 19.5 | 15.1 | 21.9 | 23.9 | 19.8 | 20.2 |
|  | More than 54 | 31.6 | 21.3 | 34.0 | 31.2 | 18.0 | 30.2 | 28.3 |
| Households with: | Children under 12 years | 37.5 | 48.6 | 25.9 | 24.8 | 35.2 | 30.7 | 34.1 |
|  | Adults over 70 years | 10.4 | 8.7 | 11.9 | 8.4 | 11.7 | 7.9 | 9.6 |
| Education level | Not completed | 1.8 | 0.9 | 0.8 | 4.1 | 0.4 | 2.9 | 2.0 |
|  | Elementary studies | 7.3 | 2.8 | 5.8 | 16.4 | 1.8 | 6.4 | 7.2 |
|  | Secondary studies | 45.6 | 50.4 | 50.9 | 42.6 | 55.3 | 43.8 | 47.3 |
|  | University | 45.2 | 46.0 | 42.6 | 36.9 | 42.5 | 46.9 | 43.5 |
| Current financial situation | Difficult | 14.3 | 12.5 | 11.8 | 19.7 | 17.6 | 14.9 | 15.0 |
|  | Regular | 51.0 | 51.9 | 48.4 | 47.8 | 49.4 | 42.6 | 48.5 |
|  | Good | 23.8 | 24.5 | 30.8 | 21.5 | 18.9 | 32.0 | 25.6 |
|  | Very good | 11.0 | 11.1 | 9.0 | 10.9 | 14.1 | 10.5 | 10.9 |

Table 2: New Ecological Paradigm (NEP) reduced scale.

| NEP Statements |
| --- |
| 1. The balance of nature is strong enough to deal with the impact caused by economic development |
| 1. Over time, humans can learn how nature works to be able to control it |
| 1. Human ingenuity will ensure that we do not make the earth an uninhabitable place |
| 1. Humans have the right to modify the environment to adapt it to their needs |
| 1. Plants and animals have as much right to exist as humans |
| 1. The balance of nature is very delicate and easily alterable |
| 1. If things continue as they are, we will soon face a major ecological catastrophe |
| 1. Despite our special abilities, humans are still dependent on the laws of nature |

Table 3: Latent dimensions identified as “ecocentric” and “anthropocentric” attitudes

|  | **Belgium** | | | **Croatia** | | | **Hungary** | | | **Italy** | | | **Poland** | | | **Spain** | | | | **Global** | | |
| --- | --- | --- | --- | --- | --- | --- | --- | --- | --- | --- | --- | --- | --- | --- | --- | --- | --- | --- | --- | --- | --- | --- |
|  | **Component** | | | **Component** | | | **Component** | | | **Component** | | | **Component** | | | **Component** | | | | **Component** | | |
|  | **1** | **2** | | **1** | **2** | | **1** | **2** | | **1** | **2** | | **1** | **2** | | **1** | **2** | | **1** | | **2** |  |
| **Q1.** | -0.24 | 0.73 | | -0.25 | 0.67 | | -0.31 | 0.66 | | -0.29 | 0.70 | | -0.14 | 0.73 | | -0.17 | 0.74 | | -0.23 | | 0.71 |  |
| **Q2.** | 0.19 | 0.72 | | 0.14 | 0.73 | | 0.11 | 0.84 | | 0.07 | 0.80 | | 0.21 | 0.76 | | 0.10 | 0.78 | | 0.12 | | 0.78 |  |
| **Q3.** | 0.08 | 0.77 | | 0.07 | 0.74 | | 0.22 | 0.79 | | 0.07 | 0.78 | | 0.15 | 0.69 | | 0.07 | 0.78 | | 0.13 | | 0.76 |  |
| **Q4.** | -0.24 | 0.71 | | -0.40 | 0.59 | | -0.38 | 0.56 | | -0.36 | 0.66 | | -0.23 | 0.67 | | -0.24 | 0.70 | | -0.31 | | 0.65 |  |
| **Q5.** | 0.73 | -0.02 | | 0.75 | -0.06 | | 0.75 | -0.03 | | 0.74 | -0.05 | | 0.78 | 0.00 | | 0.81 | -0.05 | | 0.76 | | -0.03 |  |
| **Q6.** | 0.65 | 0.01 | | 0.78 | -0.01 | | 0.83 | -0.03 | | 0.82 | -0.10 | | 0.66 | 0.10 | | 0.85 | -0.06 | | 0.77 | | -0.03 |  |
| **Q7.** | 0.77 | -0.18 | | 0.78 | -0.12 | | 0.83 | -0.14 | | 0.81 | -0.14 | | 0.82 | -0.14 | | 0.82 | -0.08 | | 0.81 | | -0.13 |  |
| **Q8.** | 0.76 | 0.03 | | 0.71 | -0.02 | | 0.84 | 0.06 | | 0.81 | -0.04 | | 0.81 | 0.01 | | 0.80 | -0.02 | | 0.80 | | 0.00 |  |
| **Explained variance by component %** | | | | | | | | | | | | | | | | | | | | | | |
|  | 28.6 | 27.3 | | 31.5 | 23.6 | | 36.8 | 26.4 | | 34.4 | 27.7 | | 31.5 | 25.7 | | 34.6 | 28.4 | | 33.2 | | 26.7 |  |
| **Total Explained variance** | | | | | | | | | | | | | | | | | | | | | | |
|  | 55.9% | | | 55.1% | | | 63.2% | | | 62.1% | | | 57.7% | | | 63.0% | | | | 59.8% | | |
| **(KMO) Kaiser-Meyer-Olkin Test** | | | | | | | | | | | | | | | | | | | | | | |
|  | 0.751 | | | 0.777 | | | 0.778 | | | 0.812 | | | 0.758 | | | 0.794 | | | | 0.790 | | |
| **Bartlett Test (significance)** | | | | | | | | | | | | | | | | | | | | | | |
|  | 1662.34 | | 816.35 | | | 2655.82 | | | 1891.10 | | | 1923.62 | | | 2656.45 | | | 11239.87 | | | | |
| **Cronbach Alfa** | | | | | | | | | | | | | | | | | | | | | | |
|  | 0.676 | | 0.687 | | | 0.726 | | | 0.763 | | | 0.638 | | | 0.733 | | | 0.687 | | | | |

References

1. Chander, M., Bodapati, S., Mukherjee, R., & Kumar, S. (2011). Organic livestock production: an emerging opportunity with new challenges for producers in tropical countries. *Rev. sci. tech. Off. int. Epiz*., 30 (3), p 569-583.
2. Sundrum, A. (2001). Organic livestock farming: acritical review. *Livestock Production Science*, 67(3), 207-215.
3. Slagboom, M., Kargo, M., Edwards, D., Sørensen, A.C., Thomasen,J.R.,& Hjortø, L. (2016). Organic dairy farmers put more emphasis on production traits than conventional farmers. *Journal of Dairy Science*, 99 (12), 9845-9856.
4. Van Wagenberg C. P. A., de Haas Y., Hogeveen H., van Krimpen M. M., Meuwissen M. P. M., van Middelaar C. E., Rodenburg T. B., (2017) Animal Board Invited Review: Comparing conventional and organic livestock production systems on different aspects of sustainability, *Animal*, (11), 10, p1839-1851, https://doi. org/10. 1017/S175173111700115X.
5. Nowak, B., Nesme, T., David, C., & Pellerin, S. (2013). To what extent does organic farming rely on nutrient in flows from conventional farming? *Environmental Research Letters*, 8 (4), 044045.
6. Läpple, D. (2013). Comparing attitudes and characteristics of organic, former organic and conventional farmers: Evidence from Ireland. Renewable *Agriculture and Food Systems*, 28 (4), 329-337.
7. [Yu-nan Xue, Wei-xin Luan, Hui Wang, Yu-jie Yang, (2019). Environmental and economic benefits of carbon emission reduction in animal husbandry via the circular economy: Case study of pig farmingin Liaoning, China, *Journal of Cleaner Production*, (238). 117968, https://doi.org/10.1016/j.jclepro.2019.117968.](file:///C:\Users\creda\Google%20Drive\_work%20creda-upc\_Proyetos\2.Nutri2cycle\n2c%20data\MODELO%20CH-EXP\_informe\old\Yu-nan%20Xue,%20Wei-xin%20Luan,%20Hui%20Wang,%20Yu-jie%20Yang,%20(2019).%20Environmental%20and%20economic%20benefits%20of%20carbon%20emission%20reduction%20in%20animal%20husbandry%20via%20the%20circular%20economy:%20Case%20study%20of%20pig%20farmingin%20Liaoning,%20China,%20Journal%20of%20Cleaner%20Production,%20(238).%20117968,%20https:\doi.org\10.1016\j.jclepro.2019.117968.)
8. Koppelmäki, K., Helenius, J., & Schulte, R. P. (2021). Nested circularity in food systems: a Nordic case study on connecting biomass, nutrient and energy flows from field scale to continent. *Resources, Conservation and Recycling*, *164*, 105218. https://doi.org/10.1016/j.resconrec.2020.105218
9. Qiong Yue, Ping Guo, Hui Wu, Youzhi Wang, Chenglong Zhang, (2022). Towards sustainable circular agriculture: An integrated optimization framework for crop-livestock-biogas-crop recycling system management under uncertainty, *Agricultural Systems*, (196), 103347, https://doi. org/10. 1016/j. agsy. 2021. 103347.
10. Rodias, E., Aivazidou, E., Achillas, C., Aidonis, D., & Bochtis, D. (2020). Water-energy-nutrients synergies in the agrifood sector: *Acircular economy framework*. Energies, 14(1), 159.
11. Vlachokostas, C., Achillas, C., Diamantis, V., Michailidou, A.V., Baginetas, K., &Aidonis, D. (2021). Supporting decision making to achieve circularity via a biodegradable waste-to-bioenergy and compost facility. *Journal of Environmental Management*, 285, 112215.
12. Puech, C., Baudry, J., Joannon, A., Poggi, S., & Aviron, S. (2014). Organic vs. Conventional farming dichotomy: does it make sense for natural enemies? *Agriculture, Ecosystems & Environment*, 194, 48-57.
13. Tagarakis, A.C.; Dordas, C.; Lampridi, M.; Kateris, D.; Bochtis, D. (2021). A Smart Farming System for Circular Agriculture. Eng. Proc. 2021, 9, 10. https://doi.org/10.3390/engproc2021009010
